# Gamma-tocopherol-*N,N*-dimethylglycine ester as a potent post-irradiation mitigator against whole body X-irradiation-induced bone marrow death in mice

**DOI:** 10.1093/jrr/rrt094

**Published:** 2013-08-01

**Authors:** Kazunori Anzai, Megumi Ueno, Ken-ichiro Matsumoto, Nobuo Ikota, Jiro Takata

**Affiliations:** 1 Nihon Pharmaceutical University; 2 National Institute of Radiological Sciences; 3 School of Pharmacy, Shujitsu University; 4 Faculty of Pharmaceutical Sciences, Fukuoka University

**Keywords:** vitamin E analog, radiation mitigator, bone marrow death, X-ray, whole body irradiation

## Abstract

We examined the radioprotective and mitigative effects of gamma-tocopherol-*N,N*-dimethylglycine ester (GTDMG), a novel water-soluble gamma-tocopherol derivative, against X-irradiation-induced bone marrow death in mice. Mice (C3H, 10 weeks, male) were injected intraperitoneally with GTDMG suspended in a 0.5% methyl cellulose solution before or after receiving of 7.5-Gy whole body X-irradiation. GTDMG significantly enhanced the 30-day survival rate when given 30 min before or immediately after the irradiation. Its mitigative activity (administered after exposure) was examined further in detail. The optimal concentration of GTDMG given immediately after irradiation was around 100 mg/kg body weight (bw) and the 30-day survival rate was 97.6 ± 2.4%. When GTDMG was administered 1, 10 and 24 h post-irradiation, the survival rate was 85.7 ± 7.6, 75.0 ± 9.7 and 36.7 ± 8.8%, respectively, showing significant mitigation even at 24 h after irradiation (*P* < 0.05). The value of the dose reduction factor (100 mg/kg bw, given intraperitoneally (i.p.) immediately after irradiation) was 1.25. GTDMG enhanced the recovery of red blood cell-, white blood cell-, and platelet-counts after irradiation and significantly increased the number of endogenous spleen colonies (*P* < 0.05). Subcutaneous (s.c.) administration also had mitigative effects. In conclusion, GTDMG is a potent radiation mitigator.

## INTRODUCTION

Research into radiation modifiers has been revived for two reasons. The first is a drastic improvement in radiation therapy for cancer. For treatments to be effective, the target tissue must be irradiated with a sufficient dose, which increases the risk of damage to surrounding normal tissues. Finding ways to reduce this risk is fundamental to improving the outcome of radiation therapy. Using chemicals that modify the effects of radiation is one way to lessen damage to normal tissues, and various radioprotective compounds have been reported [1–4]. Such compounds are called ‘radiation protectors’, and are administered pre-irradiation. Although many compounds have been reported, amifostine (WR-2721) is the only drug approved for clinical use by FDA in the USA. It is used for patients of head and neck cancer to reduce xerostomia when receiving radiotherapy. The dose reduction factor (DRF) of amifostine [500 mg/kg, intraperitoneal administration] has been reported to be 2.7 [5]. The second reason is a recent increase in the risk of accidental overexposure [6, 7]. Agents which reduce radiation damage in individuals unintentionally exposed through accidents can obviously only be given after the exposure has occurred. Such agents are called ‘radiation mitigators’. However, relatively few of these agents have been reported to date [8–20], although the number of such agents is increasing. We have reported a zinc-containing heat-treated yeast powder [21] and a water-soluble alpha-tocopherol analog (alpha-tocopherol-mono-glucoside) [22] as radiation mitigators.

Naturally occurring vitamin E is a family of eight molecules, which are characterized by a chromanol ring structure and a side chain containing two methyl groups in the middle and two more methyl groups at the end [23]. Alpha-tocopherol is one of the isomers and its radioprotective action has been reported [24–27], although some authors have not been able to demonstrate its radiation protective activity [28, 29]. Post-irradiation administration of alpha-tocopherol is reportedly consistent in effectiveness against radiation-induced death of mice [19, 20, 30, 31]. Gamma-tocotrienol and delta-tocotrienol, other type of isomers, have also been reported as a radiation protector and radiation mitigator, respectively [32–37].

**Fig. 1.**
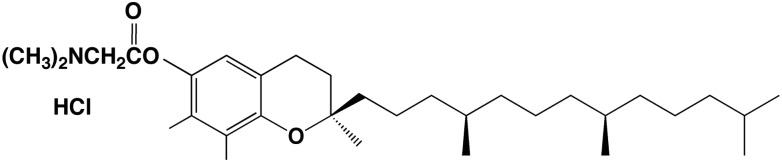
Structure of gamma-tocopherol-*N,N*-dimethylglycine ester (GTDMG).

A unique compound, gamma-tocopherol-*N,N*-dimethylglycine ester (GTDMG, Fig. 1), was developed as a novel water-soluble vitamin E derivative [38]. GTDMG acts as a pro-drug of gamma-tocopherol. GTDMG and its major metabolite, 2,7,8-trimethyl-2*S*-(beta-carboxyethyl)-6-hydroxylchroman, have received attention concerning their unique pharmacological activities. The present study aimed to evaluate the preventive effects of GTDMG against bone marrow death of mice when administered post-irradiation. GTDMG showed a remarkable enhancement of survival when administered after X-irradiation, suggesting that it may be useful for ameliorating the effects of radiation following accidental overexposure.

## MATERIALS AND METHODS

### Materials

GTDMG was synthesized as described previously [38]. Other reagents were of analytical grade and used without further purification. The white powder of GTDMG was pulverized finely with a mortar and pestle. A 0.5% methylcellulose solution (MC, methylcellulose 50, Wako Ltd., Osaka, Japan) was added to the powder of GTDMG, and the suspension was mixed well. The turbid suspension was directly administered i.p. to mice.

### Animals

The mice used in the present study were treated and handled according to the Recommendations for the Handling of Laboratory Animals for Biomedical Research compiled by the Committee for Safety and Handling Regulations for Laboratory Animal Experiments in the National Institute of Radiological Sciences (NIRS). We used male C3H mice for the experiments, because the NIRS has accumulated data on the effect of radiation on C3H mice, and we have used male C3H mice for our earlier experiments on radioprotective agents. The mice were obtained from Japan SLC Co. (Hamamatsu, Japan) at 8 weeks of age. They were housed five per cage and allowed free access to a commercial diet (MB-1, Funabashi Farm Co., Funabashi, Japan) and acidified water (pH 3.0 ± 0.2) during the experimental period. The animal rooms were maintained on a 12-h light–dark cycle, at an air temperature of 23 ± 1°C, and a humidity of 55 ± 5%. The mice were 65–75 d old at the time of irradiation and weighed 24–29 g.

### X-irradiation of mice

Each mouse was weighed and an average weight was calculated for each injection group. Usually, a 0.3-ml volume of the GTDMG suspension in 0.5% MC was administered intraperitoneally (i.p.) or subcutaneously (s.c.) before or after X-irradiation. A group of 10 mice were transferred to a round Lucite container (12 rooms, 23 cm in diameter, 4 cm high). The container was placed on the stand of an irradiator (Pantak HF-320, Shimadzu, Kyoto, Japan), and the mice were irradiated with X-rays at 200 kV and 20 mA with a filter (0.5 mm Cu/0.5 mm Al). The radiation dose was determined with a dose meter placed in a compartment of the container. The dose rate used was ∼ 0.55 Gy/min. Throughout the experiments, the radiation dose was set at 7.5 Gy except where otherwise stated in the figure legends. After irradiation, the mice were separated into groups of 5 and assessed daily for survival for 30 d.

### Administration of GTDMG

The turbid suspension of GTDMG was directly administered to mice i.p. or s.c. The dose of GTDMG was usually 100 mg/kg unless otherwise stated. Based on the experimental changing of the dose of GTDMG, no acute toxicity was observed, even at the dose of 1000 mg/kg body weight (bw) (data not shown).

### Hematological examination of peripheral blood

A sublethal radiation dose (6.0 Gy) was used in the hematological examination to ensure that mice survived for the duration of the experiment. Five groups (total 83 mice) were used, as shown in Table 1. Group A: sham irradiation with no injection. Group B: sham irradiation and i.p. injection with the vehicle (0.5% MC). Group C: sham irradiation and i.p. injection with GTDMG (100 mg/kg bw). Group D: X-irradiation and i.p. injection with the vehicle. Group E: X-irradiation and i.p. injection with GTDMG. The GTDMG suspension or vehicle solution was i.p. injected immediately after the irradiation. The initial samples of blood corresponding to time 0 were collected only for group A (*n* = 5). Other samples were collected at Days 14 and 28 for groups B and C, and at Days 3, 7, 14, 21 and 28 for groups D and E. Mice were anesthetized with diethyl ether and the blood was collected from the drain of venous blood from a cut in the armpit. The blood was transferred immediately into EDTA-treated tubes (Sysmex Co., Kobe, Japan). Total red blood cells (RBCs), white blood cells (WBCs), platelets (PLTs), neutrophils, and lymphocytes were counted with an automated hematology analyzer (SF-3000, Sysmex Co., Kobe, Japan) attached to an analyzing unit for animals (SFVU-1, Sysmex Co., Kobe, Japan).

**Table 1.** Schedule for the hematological examination

Sampling Schedule						
(Days after irradiation)	0	3	7	14	21	28
Group A	5					
Group B				5		5
Group C				5		5
Group D		5	6	6	6	6
Group E		5	6	6	6	6

The values are numbers of mice used. Group A: Sham irradiation + no injection, Group B: Sham irradiation + vehicle, Group C: Sham irradiation + γTDMG, Group D: 6 Gy + vehicle, Group E: 6 Gy + γTDMG.

### Endogenous spleen colony assay

The number of endogenous spleen colonies formed after X-irradiation was measured as reported previously [39]. Mice (five per group) were administered i.p. with the GTDMG suspension or control solution (methylcellulose or saline) immediately after receiving 7.5 Gy of whole body X-irradiation. The mice were sacrificed at Day 11 post-irradiation, and the spleens were removed. They were weighed and fixed in Bouin's solution. The number of colonies on the surface of the spleens was counted under a stereomicroscope (SZ-STU1, Olympus, Tokyo, Japan).

### Statistical analysis

Data were statistically analyzed using the software GraphPad Prism (GraphPad Software Inc., La Jolla, CA, USA). For the survival data, Kaplan–Meier plots were analyzed with a log-rank test. For the spleen colony assay data, an unpaired *t*-test was used. Differences were considered statistically significant at *P* < 0.05.

## RESULTS

**Fig. 2.**
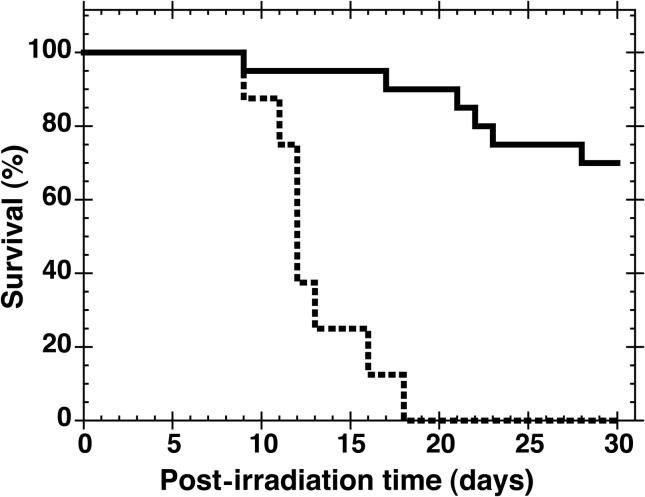
Survival curves of mice after 7.5 Gy whole body X-irradiation. Vehicle (0.3 ml of 0.5% methylcellulose solution, broken line, *n* = 8) or 100 mg/kg bw GTDMG (solid line, *n* = 20) was i.p. injected 30 min before the irradiation. The survival rate of GTDMG-administered mice was 70 ± 10% (*n* = 20).

As shown in Fig. 2, GTDMG (100 mg/kg bw) administered i.p. 30 min before whole body X-irradiation at a near lethal dose (7.5 Gy) significantly protected mice from bone marrow death (*P* < 0.05). The survival rate of mice administered GTDMG was 70 ± 10% (*n* = 20).

**Fig. 3.**
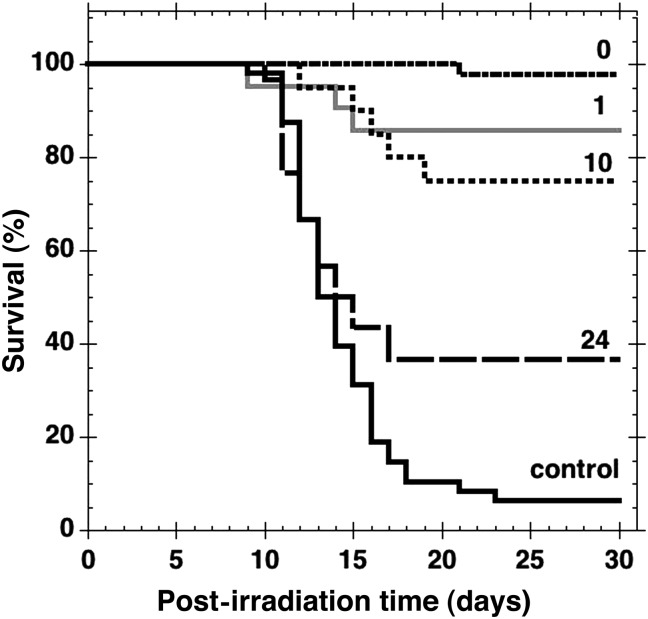
Mitigative activity of GTDMG (100 mg/kg bw) given i.p. to mice after 7.5 Gy whole body X-irradiation. The mice were administered i.p. with GTDMG at different times after the exposure. The numbers 0, 1, 10 and 24 indicate the administration immediately, 1 h, 10 h and 24 h after irradiation, respectively. The survival rate was 98 ± 2.4% (*n* = 42), 86 ± 7.6% (*n* = 21), 75 ± 9.7% (*n* = 20), and 37 ± 8.8% (*n* = 30), respectively. Vehicle solution (MC) was administered i.p. for the control group immediately after irradiation (*n* = 48).

When GTDMG (100 mg/kg bw) was administered immediately after the irradiation, the survival curve indicated a potent reduction in bone marrow death with a 30-d survival rate of 97.6 ± 2.4% (*n* = 42) (Fig. 3, the curve indicated as 0). Since the results suggested that GTDMG is a radiation mitigator, the timing of its administration was varied and the survival rate of mice was measured. As shown in Fig. 3, the survival rate was highest when GTDMG was given i.p. immediately after irradiation. The survival rate decreased gradually as the duration between the irradiation and administration increased. When GTDMG was administered at 1 h, 10 h and 24 h post-irradiation, the survival rate at Day 30 was 85.7 ± 7.6% (*n* = 21), 75.0 ± 9.7% (*n* = 20), and 36.7 ± 8.8% (*n* = 30), respectively. Even following the administration at 24 h after exposure, the log-rank test showed the survival curve to be significantly different to that of the control (*P* < 0.05).

**Fig. 4.**
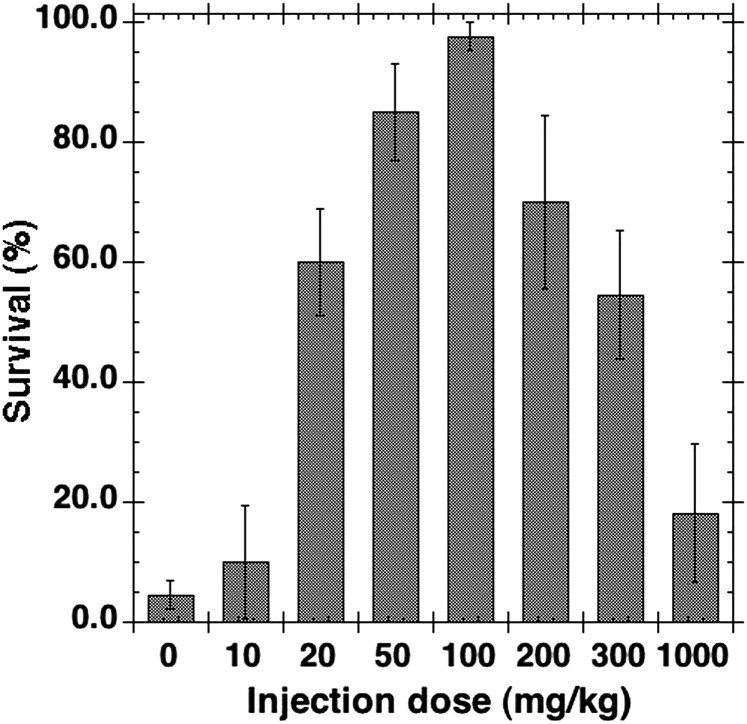
The 30-d survival rate of mice injected with various doses of GTDMG immediately after exposure to 7.5 Gy of X-irradiation. The number of mice used was 66, 10, 30, 20, 42, 10, 21 and 11 for the injection dose of 0 (= control), 10, 20, 50, 100, 200, 300 and 1000 mg/kg bw, respectively.

Figure 4 shows the survival rate of mice 30 d post-irradiation obtained while changing the dose of GTDMG. GTDMG was administered immediately after whole body irradiation with 7.5-Gy X-rays. The survival rate shows a bell-shaped dependency on the dose of GTDMG. The survival rates of mice injected with 20, 50, 100, 200 and 300 mg/kg bw were significantly higher than that of the control. The maximum effect was observed at 100 mg/kg bw but this was not significantly different from the rate observed at 50 mg/kg bw. The administration of doses higher than 100 mg/kg bw resulted in a significantly reduced effect. The survival rates of mice injected with 10 and 1000 mg/kg bw were not significantly different from that of the control.

**Fig. 5.**
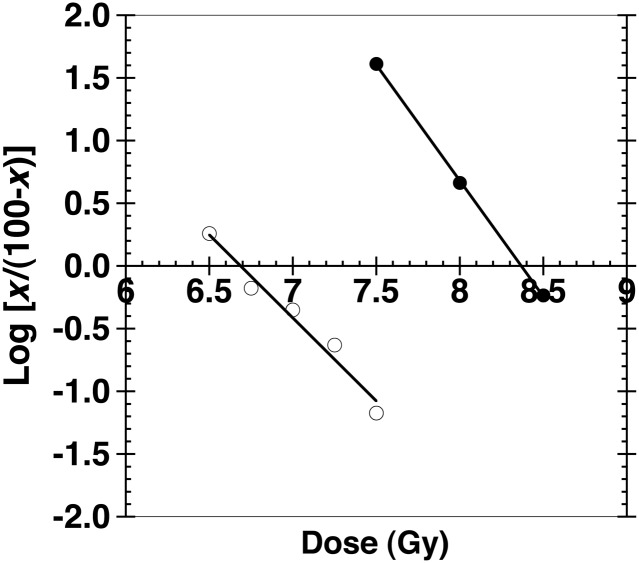
Probit plot of the survival rate at Day 30. Closed circles are the data for the mice administered GTDMG (100 mg/kg bw) immediately after whole body X-irradiation. The number of mice used was 42, 28 and 19 for the irradiation with 7.5, 8.0 and 8.5 Gy, respectively. Open circles are the data for the control mice (administered the vehicle, a 0.5% methyl cellulose solution). The number of mice used was 17, 30, 81, 20 and 334 for 6.5, 6.75, 7.0, 7.25 and 7.5 Gy, respectively. The ordinate values were calculated using the equation log[*x*/(100 – *x*)], where *x* is the survival rate expressed as a percentage. The line at 0 shows 50% survival and the crossing points of the 0-line and each linear regression line show the LD_50/30_. The LD_50/30_ of mice injected with 100 mg/kg bw GTDMG was 8.37 Gy, whereas that of control mice (given vehicle only) was 6.69 Gy.

The activity of GTDMG in protecting bone marrow death caused by whole body irradiation was estimated as a DRF. GTDMG was given i.p. immediately after X-irradiation at various doses. As shown in Fig. 5, the LD_50/30_ of mice injected with 100 mg/kg bw GTDMG was 8.37 Gy, whereas that of control mice (administration of vehicle only) was 6.69 Gy. From these two values, the DRF of GTDMG (100 mg/kg bw, i.p. administration immediately after irradiation) was calculated to be 1.25.

**Fig. 6.**
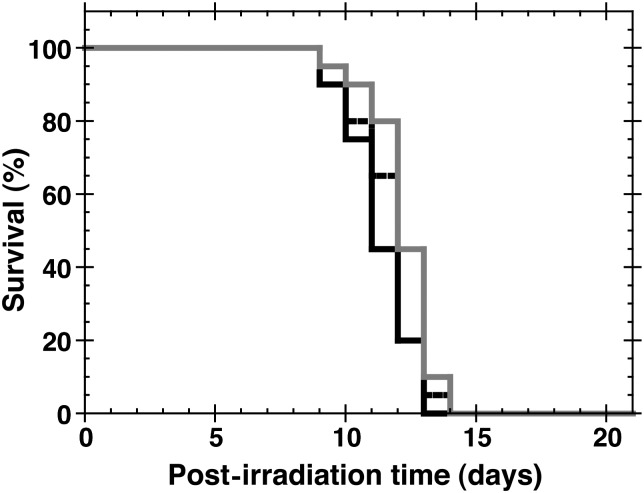
Effect of dimethylglycine or gamma-tocopherol on the survival of mice administered after exposure to X-rays. Mice were whole body irradiated with 7.5 Gy X-rays and dimethylglycine in MC (25.9 mg/kg bw) or gamma-tocopherol in MC (77.4 mg/kg bw) was administered i.p. immediately after irradiation. Vehicle solution (MC) was used for the control. Black solid line, gray solid line, and black dotted line show the data for the group of mice injected with vehicle (MC), gamma-tocopherol, and dimethylglycine, respectively. The black dotted line almost overlaps with the other two lines. Number of mice used was 20 for each group.

When dimethylglycine, the concentration of which corresponded to 100 mg/kg bw GTDMG, was i.p injected immediately after 7.5-Gy whole body irradiation (*n* = 20), no significant extension of survival compared with the MC-injected group (*n* = 20) was observed (hazard ratio = 0.4774, *P* = 0.0915), while the mice injected with gamma-tocopherol, the concentration of which corresponded to 100 mg/kg bw GTDMG suspended in 0.5% MC (*n* = 20), showed only a small mitigative effect compared to the vehicle-injected control group (hazard ratio = 0.3573, *P* = 0.0205) (Fig. 6).

**Fig. 7.**
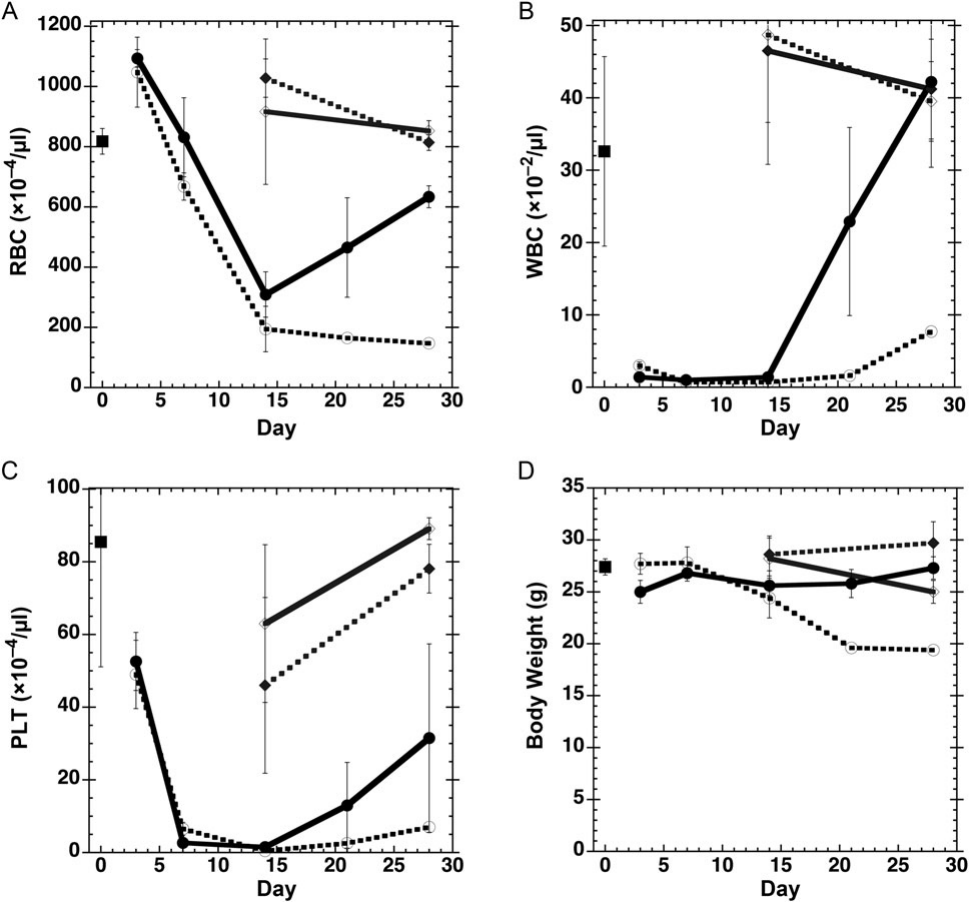
Change in the number of (**A**) red blood cells (RBC), (**B**) white blood cells (WBC), and (**C**) platelets (PLT), and (**D**) the body weight of mice. Mice were whole body irradiated with X-rays at 6.0 Gy and each parameter was measured after the irradiation for 28 d. Group A (closed squares): sham irradiation with no treatment. Group B (closed diamonds and dotted gray line): sham irradiation and treatment with vehicle (MC). Group C (open diamonds and solid gray line): sham irradiation and treatment with GTDMG. Group D (closed circles and solid black line): irradiation and treatment with GTDMG. Group E (open circles and dotted black line): irradiation and treatment with vehicle (MC).

Changes to red blood cell (RBC), white blood cell (WBC), and platelet (PLT) counts after irradiation and/or GTDMG injection until Day 28 post-irradiation are shown in Fig. 7A–C in addition to body-weight changes (Fig. 7D). The RBC count increased slightly at Day 3 and then decreased till Day 14, after which it increased for the GTDMG-treated group (group E), whereas it remained low for the vehicle-treated group (group D) (Fig. 7A). The WBC count of both the GTDMG- and vehicle-treated groups decreased drastically to almost zero at Day 3 after irradiation and remained very low till Day 14 (Fig. 7B). After Day 14, the WBC count of the GTDMG-treated group increased markedly to reach that in the un-irradiated group (group B and C), while the WBC count of the vehicle-treated group increased slightly. The PLT-count of both the GTDMG- and vehicle-treated groups decreased extensively till Day 7 and remained very low till Day 14 (Fig. 7C). The PLT count of the GTDMG-treated group recovered to the one third of the original level, while that of the vehicle-treated group recovered only slightly. Without irradiation, GTDMG did not change the RBC, WBC, or PLT count. Following irradiation, the body weight was decreased continuously from Day 7 to Day 21 in the vehicle-treated group (group D), while body weight in the GTDMG-treated group (group E) stopped decreasing and gradually recovered. Body weight in the GTDMG-treated group without irradiation (group C) was slightly lower than that in the vehicle-treated group (group B) at Day 28.

**Fig. 8.**
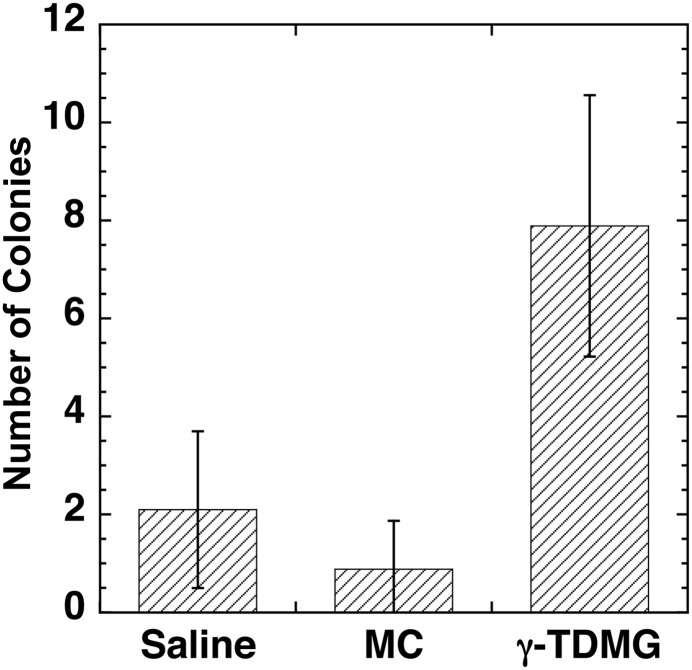
Endogenous spleen colony formation in mice treated with GTDMG, vehicle (MC), or saline after receiving 7.5-Gy whole body irradiation. Each solution was injected i.p immediately after the irradiation and the number of spleen colonies was measured at Day 11 post-irradiation.

The effect of GTDMG on the formation of endogenous spleen colonies was examined. As shown in Fig. 8, the number of colonies was significantly larger in GTDMG-treated mice at Day 11 post-irradiation than in vehicle- or saline-treated mice (*P* < 0.05).

**Fig. 9.**
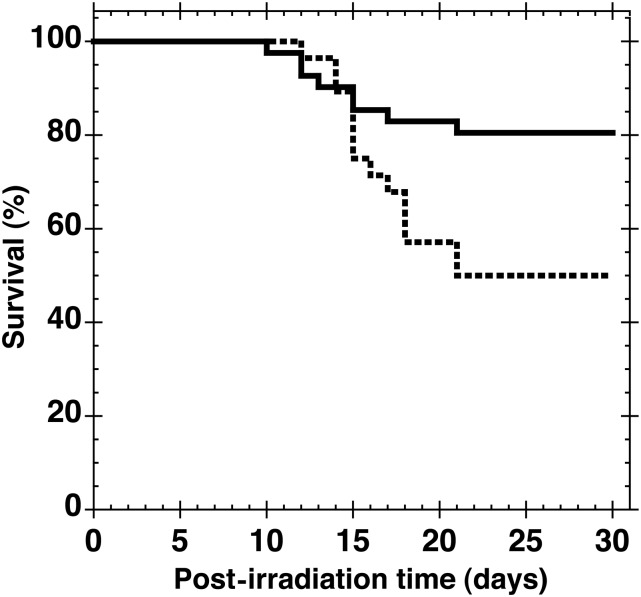
Effect of subcutaneous injection of GTDMG on the survival curves of mice after 7.5-Gy whole body X-irradiation. Vehicle (0.3 ml of 0.5% methylcellulose solution, broken line) or 100 mg/kg bw GTDMG (solid line) was s.c. injected immediately after the irradiation. The survival rate of GTDMG-injected mice was 80.5 ± 6.2% (*n* = 41), whereas that of the irradiation control group was 50.0 ± 9.4% (*n* = 28).

Finally, we examined routes of administration other than the i.p. route. As shown in Fig. 9, subcutaneous injection of GTDMG immediately after 7.5 Gy of whole body irradiation also significantly increased the 30-d survival rate (*P* < 0.05). The survival rate for the GTDMG-treated group was 80.5 ± 6.2% (*n* = 41), whereas that for the radiation control group was 50.0 ± 9.4% (*n* = 28). Oral administration of GTDMG was without effect (data not shown).

## DISCUSSION

In the present study, we showed that GTDMG, a vitamin E analog, is a strong radiation protector (given before exposure) and also a strong radiation mitigator (given after exposure) acting to prevent bone marrow death in mice. Radiation mitigators should be useful in cases of accidental overexposure but relatively few compounds have been examined. Here we studied the mitigative activity of GTDMG in detail. Only a limited number of compounds have been reported to show radiation mitigation (radioprotection when administered after irradiation), for example, the vitamin E family (19, 20), glucan [4], heat-killed *Lactobacillus casei* [15, 16], tocopherol-monoglucoside [22, 40], heat-treated mineral yeast powder [21], CBLB502 [9], CBLB613 [13], ALXN4100TPO [10] and GR1977143 [8]. In the present study, we showed that the DRF value of GTDMG was 1.25 when given at 100 mg/kg bw by the i.p. route. This value is the highest for a mitigator among vitamin E derivatives reported so far. In addition, GTDMG had a significant mitigative activity, even at 24 h post-irradiation.

GTDMG is hydrolyzed to form gamma-tocopherol and dimethylglycine. We tried to determine whether the mitigative effect is due to GTDMG itself or due to hydrolyzed gamma-tocopherol and dimethylglycine. We observed that dimethylglycine (at a concentration corresponding to 100 mg/kg bw GTDMG) i.p injected alone immediately after irradiation did not have a mitigative effect, and gamma-tocopherol injected alone had only a weak effect. Therefore, dimethylglycine is not responsible for the effect. Although this observation suggests that GTDMG itself is responsible for the mitigation, we cannot exclude the possibility that gamma-tocopherol is an active component, because it is not clear whether the concentration of gamma-tocopherol was high enough when injected alone in the MC solution. Alpha-tocopherol used in a properly solubilized form has been reported to act as a radioprotector [24–27]. Furthermore, gamma-tocotrienol and delta-tocotrienol have recently been reported as radioprotectors and mitigators [32, 33]. Experiments are in progress to measure GTDMG and gamma-tocopherol levels in plasma and bone marrow after i.p. administration. In addition to GTDMG, we observed that alpha-TDMG, alpha-tocotrienol-*N,N*-dimethylglycine ester, and gamma-tocotrienol-*N,N*-dimethylglycine ester had a significant mitigative effect (unpublished result).

GTDMG showed a bell-shaped dose dependency with maximum activity at around 100 mg/kg bw and less activity at higher concentrations. A similar tendency, i.e. that a very high concentration is less effective than the optimum concentration, was reported for the prophylactic activity of alpha-tocopherol [30] and gamma-tocotrienol [32].

The hematological examination showed that whole body irradiation decreased peripheral blood counts, but the numbers recovered significantly after post-irradiation treatment with GTDMG. Similar changes were observed following LC9018 treatment [16], gamma-tocotrienol treatment [32], and delta-tocotrienol treatment [33] after exposure. The measurement of intrinsic colony formation also showed that GTDMG enhanced proliferation. These results suggest that GTDMG enhances the recovery of peripheral blood cells. It is possible that GTDMG induces radioprotective cytokines such as G-CSF and growth factors in the way that alpha-tocopherol succinate does [41], although as yet we do not have experimental data to support this. Several other mechanisms have been considered to explain the effect of tocopherols, tocotrienols and their derivatives in their action as radiation countermeasures [33, 35–37, 42, 43]. One such mechanism is based on the antioxidant property. Although GTDMG has antioxidant activity (unpublished result), the activity may not be responsible for the mitigative effect shown in this study (antioxidant activity is usually correlated with radioprotective effect). Other mechanisms, such as inhibition of 3-hydroxy-3-methylglutaryl-coenzyme A (HMG-CoA) reductase, modulation of the expression of antioxidative enzymes, inhibition of radiation-induced apotosis, stimulation of extracellular signal-related kinase (Erk) activation-associated with the mammalian target of the rapamycin (mTOR) survival pathway, have not yet been examined with GTDMG.

Both i.p. and s.c. administration of GTDMG showed a significant mitigation effect. In contrast, oral administration of it had no significant effect on the survival of the mice (data not shown). Although we do not yet have the experimental results, it is plausible that GTDMG is hydrolyzed in the digestive tract and the resultant gamma-tocopherol (water-insoluble) is difficult to absorb in the intestine.

GTDMG has several advantages for possible practical use as a countermeasure, which are as follows: (i) a wide time window (significantly effective even at 24 h after exposure), (ii) a high DRF value compared with other known mitigators related to vitamin E, (iii) effective by s.c. injection, (iv) chemically stable and easy to handle because of its powdered form, (v) partially water-soluble, and (vi) low toxicity and immunogenicity (because it is a vitamin E analog). These features make GTDMG a good candidate for a radiation mitigator for human use.

## FUNDING

This work was supported by a Grant-in-Aid for Scientific Research (C) from the Japan Society for the Promotion of Science (22510066) and by funds from the Central Research Institute of Fukuoka University (091001).
